# Supplementary material for: Evaluation of whole genome amplification and bioinformatic methods for the characterization of Leishmania genomes at a single cell level
Source: Sci Rep. 2020 Sep 14;10:15043. doi: 10.1038/s41598-020-71882-2 (PMC7490275; doi:10.1038/s41598-020-71882-2)
Supplement: Supplementary file 1 — Supplementary file1 [file 41598_2020_71882_MOESM1_ESM.docx]

**Supplementary information**

**Evaluation of whole genome amplification and bioinformatic methods for the characterization of *Leishmania* genomes at a single cell level**

Hideo Imamura, Pieter Monsieurs, Marlene Jara, Mandy Sanders, Ilse Maes, Manu Vanaerschot, Matthew Berriman, James A. Cotton, Jean-Claude Dujardin and Malgorzata A. Domagalska

**
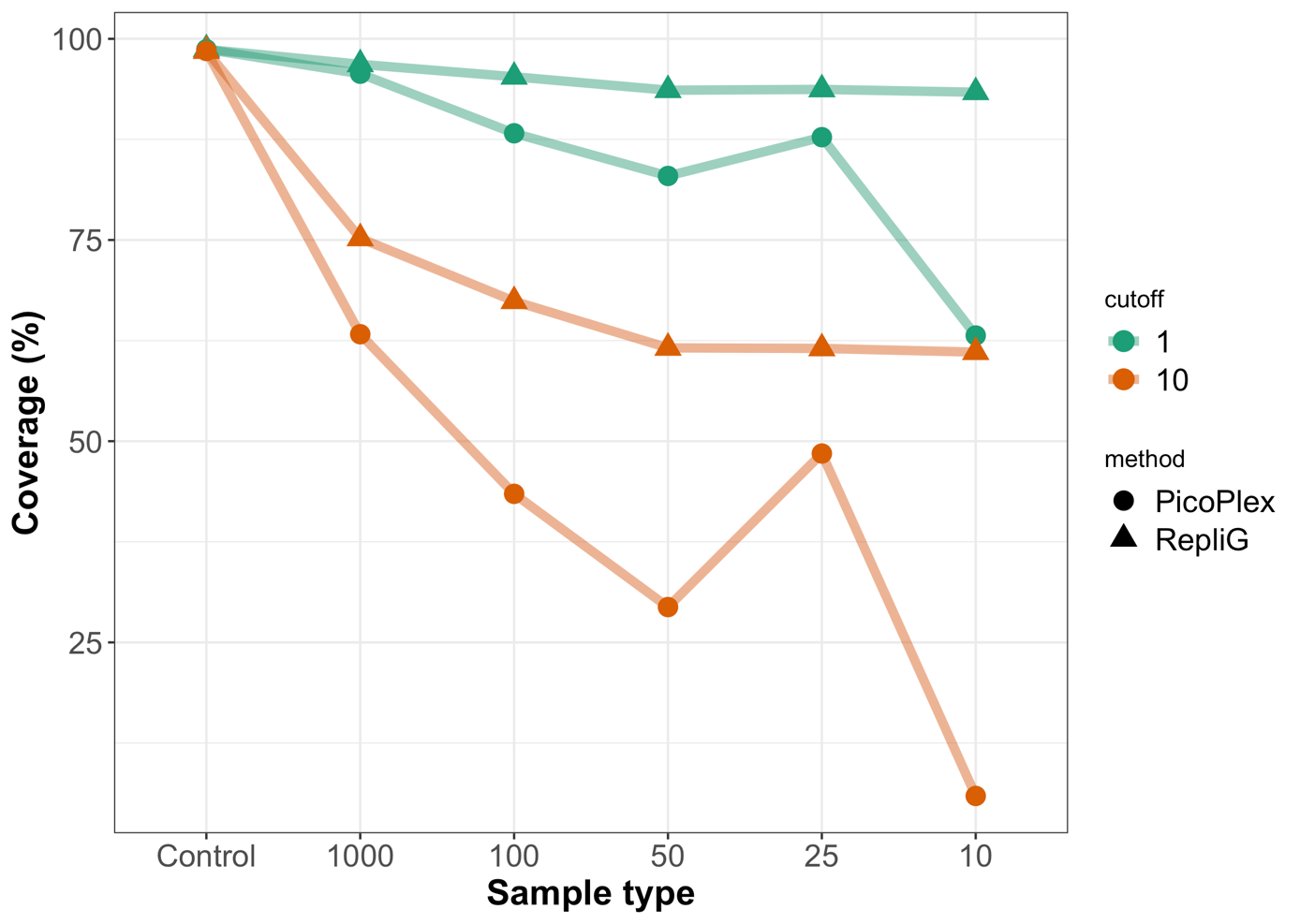
**

**Supplementary Figure S1.** For DNA extracted from different amounts of *L. donovani* BPK275 cells (1000, 100, 50, 25 or 10 cells), and amplified using RepliG or PicoPLEX, the fraction of the genome covered by at least 1 read (green line) or 10 reads (red line) is plotted. The control sample is obtained from undiluted and unamplified DNA.

**Supplementary Figure S2.** Chromosome length and GC content in reference genomes of *L. donovani (Ld)* BPK282 and *L. braziliensis* (*Lb*) M2904. The GC content was plotted for each chromosome. Correlations: (i) for *Ld*, r^2^ = 0.586, p-value 5.47e-8, (ii) for *Lb*, r^2^ = 0.566, p-value 1.89e-7.

**
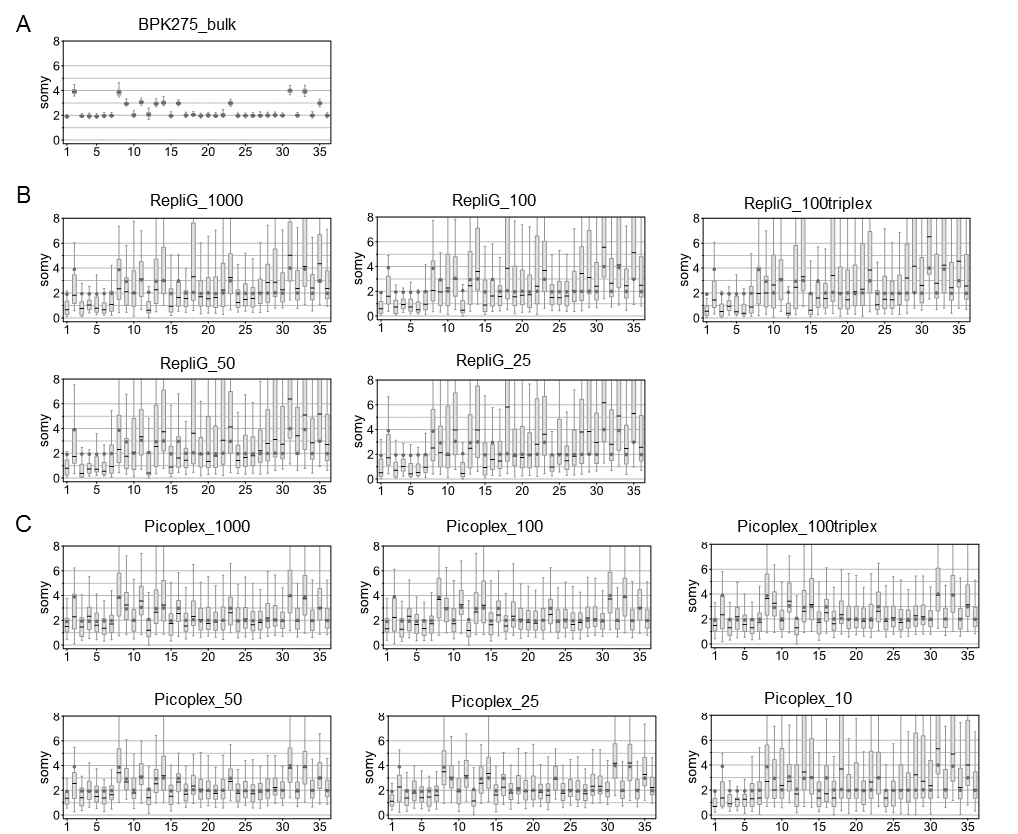
**

**Supplementary Figure S3.** Box plots for visualization of somy estimations, where a somy estimate is calculated for each 5kb window in (A) BPK275 control, (B) RepliG samples derived from different cell numbers and (C) PicoPLEX samples derived from different cell numbers. The true somy value – derived based on the BPK275 control – is given in a grey filled circle. The median somy of a chromosome in a given sample is shown as thick horizontal line.

A

**Supplementary Figure S4.** Somy visualization without (purple line) and with (green line) GC bias correction in (A) BPK275 control, (B) RepliG and (C) PicoPLEX samples. The x-axis and the y-axis represent chromosome and somy value, respectively.

**
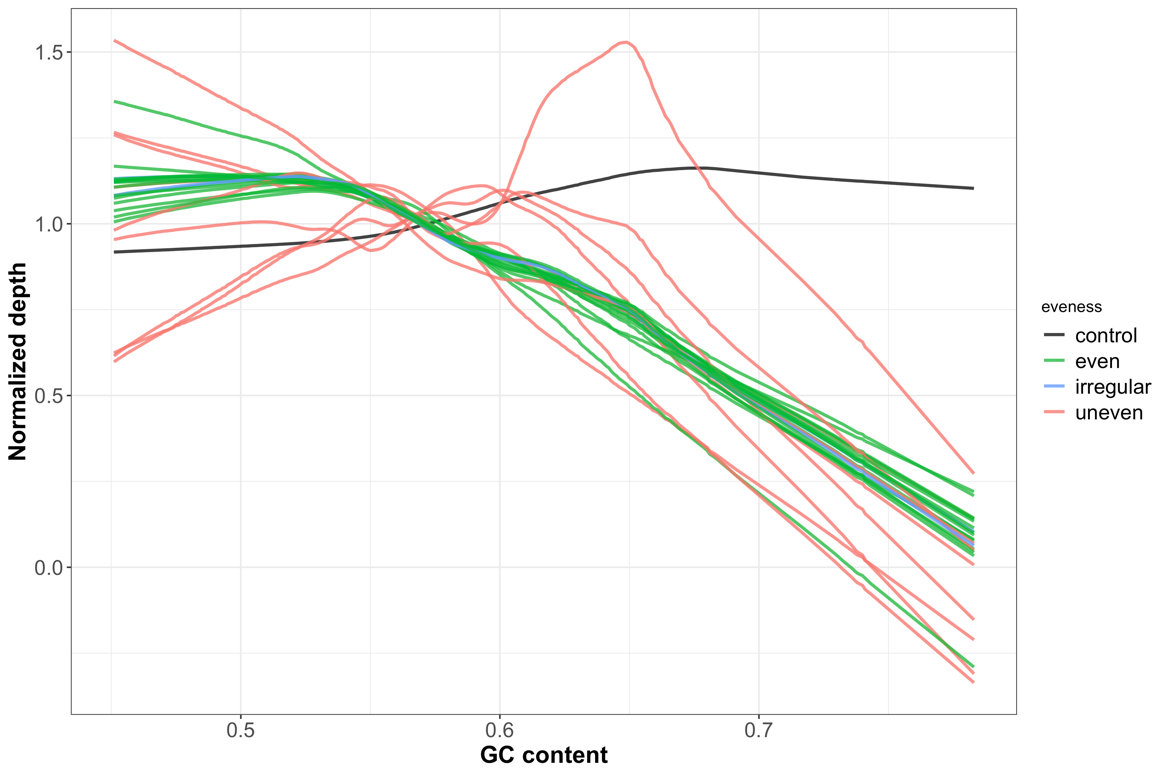
**

**
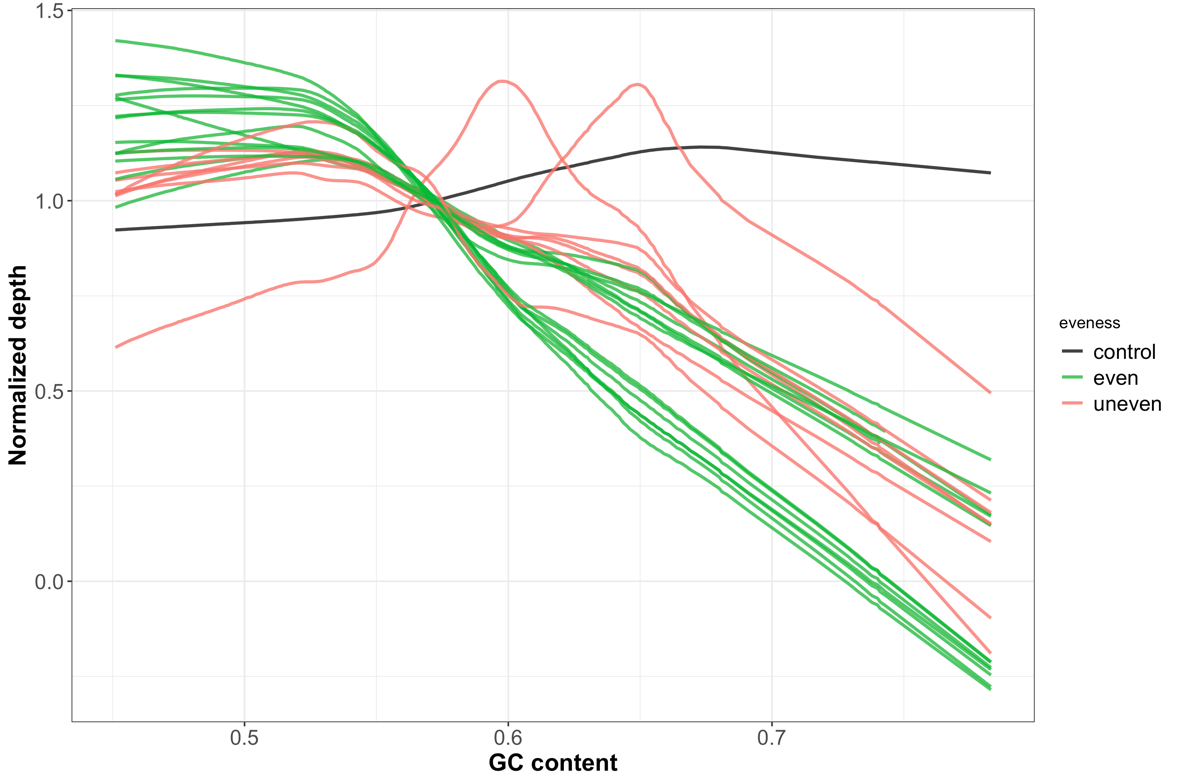
**

**Supplementary Figure S5.** Lowess curves showing the impact of GC content on normalized depth in (A) PER094a samples and (B) PER094b samples. The bulk controls are shown in black. The even, mixed and uneven depth samples were shown in light blue, light green and light brown, respectively.

**Supplementary Figure S6.** Boxplots of PER094a single cell samples: A) The first boxplot was for the PER094 control. There were 14 even depth single cells that had a short box and short error whiskers, allowing accurate somy estimation. Chromosome 1 is disomic in most samples, except PER094a_sc2, where it is monosomic, marked by the arrow. B) Mixed depth: PER093A_sc7 and PER093A_sc11 had 1 and 2 chromosomes respectively, with high variable depth. C) The uneven depth of 9 samples led to an erratic somy estimation that could not be corrected.

**Supplementary Figure S7.** Boxplots of PER094b single cell samples: A) The first boxplot was for the PER094 control. There were 14 even depth single cells that had a short box and short error whiskers, allowing accurate somy estimation. Chromosome 25 is trisomic in most samples, except PER094b_sc9 and sc2, where it is disomic, marked by the arrow. B) The uneven depth of 8 samples led to an erratic somy estimation that could not be corrected. The grey arrows indicate the disomic chromosome 25.

**
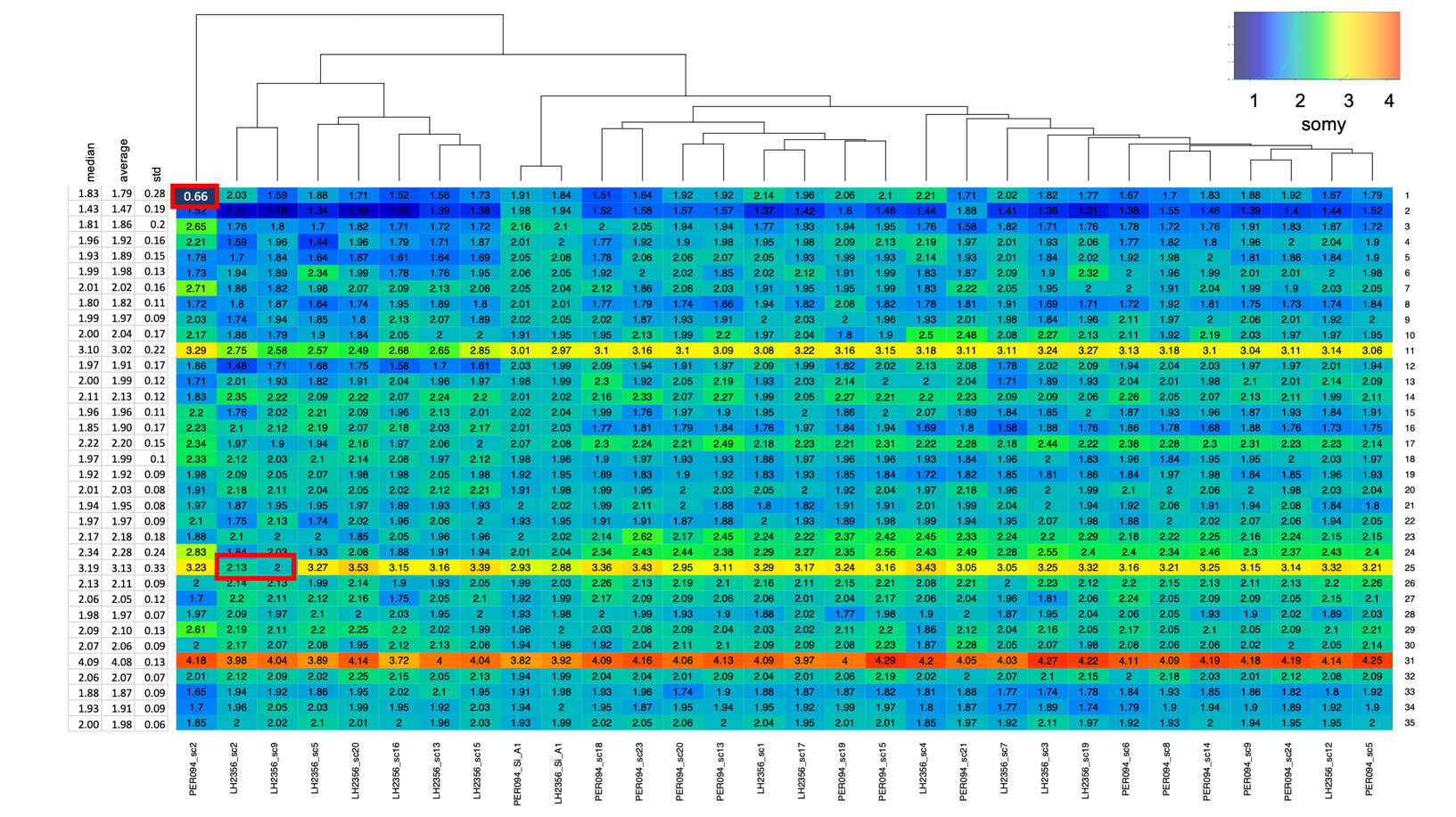
**

**Supplementary Figure S8.** Somy values with GC bias correction for 28 high quality, even depth single cells. The disomic chromosome 25 of PER094b_sc2 and PER094b_sc9 and the monosomic chromosome 1 of PER094a_sc2 are marked in a red box. The color key shows the normalized chromosome read depth, which is corresponding to GC normalized somy. The dendrogram on top of the heatmap is obtained using single-linkage hierarchical clustering, and visualizes the grouping of the cells based on their somy values.

**Supplementary Figure S9.** A) Monosomic allele frequency distribution of chromosome 1 of PER094a_sc2 was shown on the y-axis while the clock-like allele frequency distribution of PER094 control (disomic, bulk sequencing) was shown on the x-axis. Two grey arrows indicate the allele frequency shift towards 0 and 1 in PER094a_sc2. B) Disomic chromosome of PER094b_sc13 does not show a clock-like allele frequency distribution, but a flat one, probably because of allele dropouts. C) x-axis shows the bimodal allele frequency distribution of chromosome 25 in PER094 control (trisomic, bulk sequencing); y-axis, ‘flat’ allele frequency distribution in chromosome 25 of PER094b_sc9, shown to be disomic by normalized read-depth. D) y-axis, bimodal distribution of chromosome 25 in PER094b_sc15 shown to be trisomic by normalized depth. The color of dots represents the counts in log10 scale and the straight lines represent the regression lines. PD stands for probability density.

**Supplementary data**

**Supplementary_data1.xlsx** file containing 3 tables (S1 to S3)

**Supplementary Table S1.** Mapping statistics and read depth statistics for BPK275 samples were given. The summary table of somy accuracy is also given for comparison.

**Supplementary Table S2.** Mapping statistics and read depth statistics for PER094 samples were given. The summary table of somy accuracy is also given for comparison.

**Supplementary Table S3.** Impact of the median depth and normalized depth standard deviation on the somy accuracy expressed by average somy deviation (ASD). We calculated the correlation values and the p-values between these variables.

**Supplementary_data2.pdf**

Manhattan plots showing the read depth across 36 chromosomes of *L. donovani* BPK275 in (p1, A) the bulk control, (p1, B-G) PicoPLEX samples derived from different cell numbers and and (p1, H-L and p2, A) RepliG samples derived from different cell numbers. The x- and y-axes represent chromosomal position and average depth per 5000 bp, respectively.

**Supplementary_data3.pdf**

Manhattan plots showing the read depth across 35 chromosomes of *L. braziliensis* PER094a in (p1, A) the bulk control and (p1, B-L; p2, A-L; p3, A-B) PicoPLEX samples derived from different single cells. Plots are organized from higher to lower quality samples. The x- and y-axes represent chromosomal position and average depth per 5000 bp, respectively.

**Supplementary_data4 file**

Manhattan plots showing the read depth across 35 chromosomes of *L. braziliensis* PER094b in (p1, A) the bulk control and (p1, B-L; p2, A-K) PicoPLEX samples derived from different single cells. Plots are organized from higher to lower quality samples. The x- and y-axes represent chromosomal position and average depth per 5000 bp, respectively.

**Supplementary_data5.pdf**

GC uncorrected (purple) and GC normalized (green) somy values in *L. braziliensis* PER094a: (p1, A) bulk control and (p1, B-L; p2, A-L; p3, A-B) PicoPLEX samples from different single cells. Graphs are organized from higher to lower quality samples. The x- and y-axes represent chromosome numbers and somy, respectively.

**Supplementary_data6.pdf**

GC uncorrected (purple) and GC normalized (green) somy values in *L. braziliensis* PER094b: (p1, A) bulk control and (p1, B-L; p2, A-K) PicoPLEX samples from different single cells. Graphs are organized from higher to lower quality samples. The x- and y-axes represent chromosome numbers and somy, respectively.
