# Supplementary material for: Evaluation of whole genome amplification and bioinformatic methods for the characterization of Leishmania genomes at a single cell level
Source: Sci Rep. 2020 Sep 14;10:15043. doi: 10.1038/s41598-020-71882-2 (PMC7490275; doi:10.1038/s41598-020-71882-2)
Supplement: Supplementary file 2 — Supplementary file2 [file 41598_2020_71882_MOESM2_ESM.docx]

**Evaluation of whole genome amplification and bioinformatic methods for the characterization of *Leishmania* genomes at a single cell level**

Hideo Imamura, Pieter Monsieurs, Marlene Jara, Mandy Sanders, Ilse Maes, Manu Vanaerschot, Matthew Berriman, James A. Cotton, Jean-Claude Dujardin and Malgorzata A. Domagalska

**Supplementary methods**

**qPCR to verify quality of the PicoPLEX-amplified single cell samples before sequencing**

Four qPCR were used, 3 targeting *Leishmania* DNA (kDNA, 18S rDNA and G6PD) and 1 targeting human DNA (RPL30)

| **Primer** | **Sequence** | **Target** | **Amplicon**  **length (bp)** | **Ref** |
| --- | --- | --- | --- | --- |
| MP3H | 5' GAACGGGGTTTCTGTATGC 3' | kDNA minicircle | 72 | 1 |
| MP1L | 5' TACTCCCCGACATGCCTCTG 3' |  |  |  |
| 18S F | 5' CATCAAACTGTGCCGATTACGTCC  3' | 18S rDNA | 114 | 2 |
| 18S R | 5' GAACTTTCGGGCGGATAAAACACC  3' |  |  |  |
| G6PD F  G6PD R | 5’ TTGATCACTGGTACATGCATTAAG 3’  5’ CTCGTCCAGAATGCAGCAC 3’ | G6PD | 101 | 3 |
| RPL30 F | 5’ GCCCGTTCAGTCTCTTCGATT 3’ | RPL30 | 73bp | 4 |
| RPL30 R | 5’ CAAGGCAAAGCGAAATTGGTT 3’ |  |  |  |

The following cycling conditions were used:

kDNA (*Leishmania braziliensis*): 94°C for 2 mins, followed by 29 rounds of 94°C for 1 min and 54°C for 1 min

18S rDNA (*Leishmania*): 95°C for 3 min, followed by 35 cycles at 95°C for 20 s, 60°C for 20 s and 72°C for 20 s.

G6PD (*Leishmania*): 95˚C for 10 mins, followed by 45 rounds of 95˚C for 30s, 65˚C for 30s, 72˚C for 15s.

RPL30 (human): 95˚C for 10 mins, followed by 45 rounds of 95˚C for 30s, 60˚C for 15s, 72˚C for 15s.

For the QPCR, we used the Lightcycler 480 (Roche)

To select samples for whole genome sequencing we checked the following parameters :

1. Final concentration of amplified DNA as measured by nanodrop was at least 5 ng/µl
2. The Ct value for the RPL30 was 35 or higher, which is comparable to the values found in pure Leishmania DNA samples.
3. The presence of Leishmania DNA was confirmed by qPCR by at least one of the *Leishmania* markers.
4. Two samples were added based only on the amount of DNA, while the presence of Leishmania DNA was suggestive, but uncertain.

**References**

1 Lopez, M. et al. Diagnosis of *Leishmania* using the polymerase chain reaction: a simplified procedure for field work. *Am. J. Trop. Med. Hyg.* 49, 348–56 (1993).

2 Jara, M. et al. Macromolecular biosynthetic parameters and metabolic profile in different life stages of *Leishmania braziliensis*: Amastigotes as a functionally less active stage. *PLoS One* 12, e0180532 (2017).

3 Castilho, T. M., Camargo, L. M. A., McMahon-Pratt, D., Shaw, J. J. & Floeter-Winter, L. M. A real-time polymerase chain reaction assay for the identification and quantification of American Leishmania species on the basis of glucose-6-phosphate dehydrogenase. Am. J. Trop. Med. Hyg. 78, 122–132 (2008).

4 Domagalska, M. A. et al. Genomes of Leishmania parasites directly sequenced from patients with visceral leishmaniasis in the Indian subcontinent. PLoS Negl. Trop. Dis. 13, e0007900 (2019).
