# Supplementary material for: Evaluation of whole genome amplification and bioinformatic methods for the characterization of Leishmania genomes at a single cell level
Source: Sci Rep. 2020 Sep 14;10:15043. doi: 10.1038/s41598-020-71882-2 (PMC7490275; doi:10.1038/s41598-020-71882-2)
Supplement: Supplementary file 4 — Supplementary data 2 [file 41598_2020_71882_MOESM4_ESM.pdf]

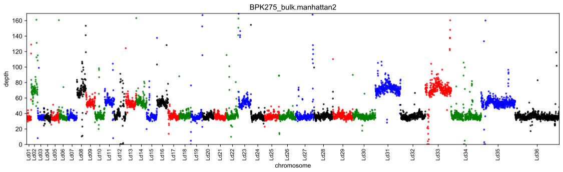

(A) BPK275_bulk

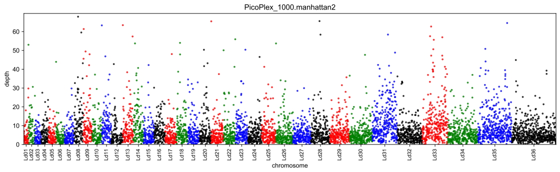

(B) PicoPlex_1000

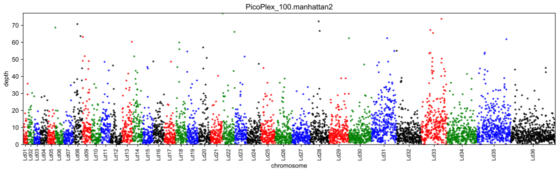

(C) PicoPlex_100

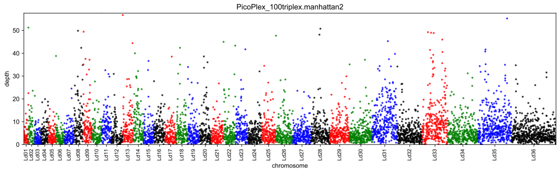

(D) PicoPlex_100triplex

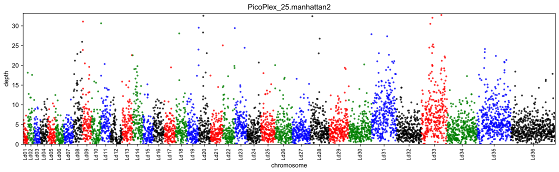

(E) PicoPlex_25

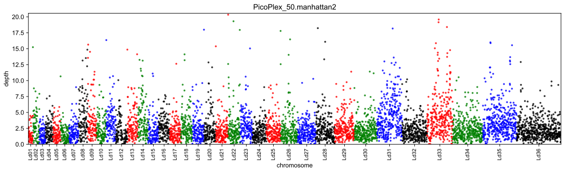

(F) PicoPlex_50

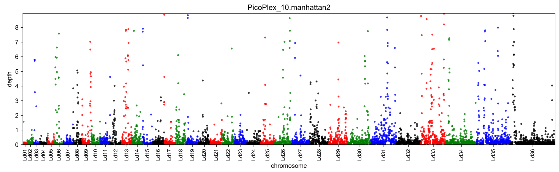

(G) PicoPlex_10

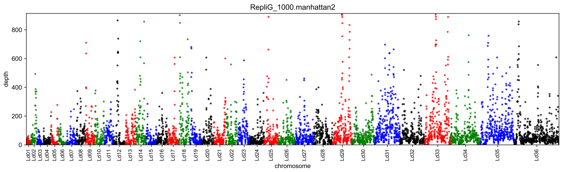

(H) RepliG_1000

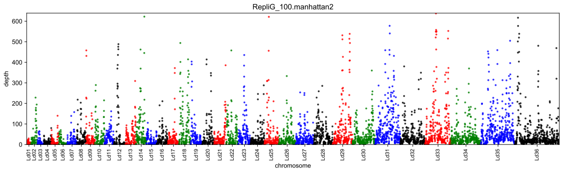

(I) RepliG_100

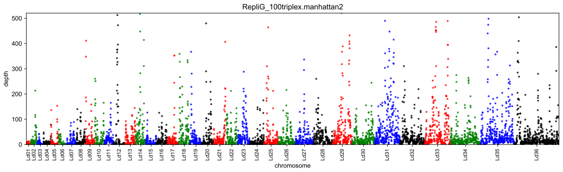

(J) RepliG_100triplex

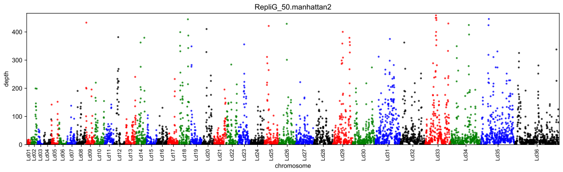

(K) RepliG_50

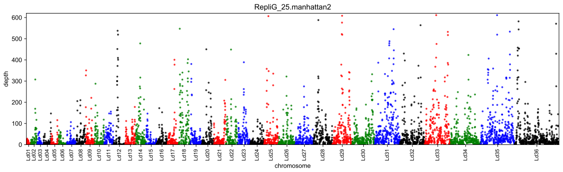

(L) RepliG_25

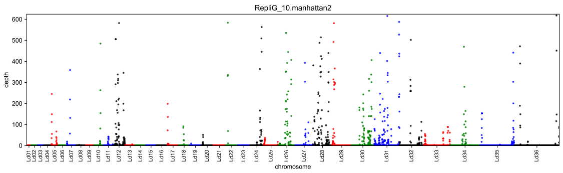
RepliG_10.manhattan2
depth
0
100
200
300
400
500
600
Ld01
Ld02
Ld03
Ld04
Ld05
Ld06
Ld07
Ld08
Ld09
Ld10
Ld11
Ld12
Ld13
Ld14
Ld15
Ld16
Ld17
Ld18
Ld19
Ld20
Ld21
Ld22
Ld23
Ld24
Ld25
Ld26
Ld27
Ld28
Ld29
Ld30
Ld31
Ld32
Ld33
Ld34
Ld35
Ld36
chromosome

(A) RepliG_10
