# Supplementary material for: Evaluation of whole genome amplification and bioinformatic methods for the characterization of Leishmania genomes at a single cell level
Source: Sci Rep. 2020 Sep 14;10:15043. doi: 10.1038/s41598-020-71882-2 (PMC7490275; doi:10.1038/s41598-020-71882-2)
Supplement: Supplementary file 7 — Supplementary data 5 [file 41598_2020_71882_MOESM7_ESM.pdf]

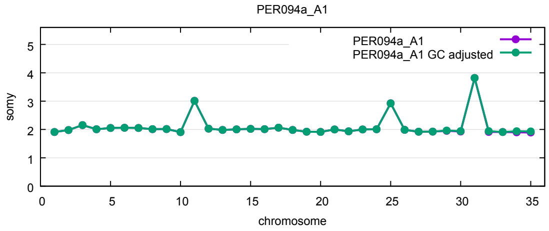

(A) PER094a_bulk

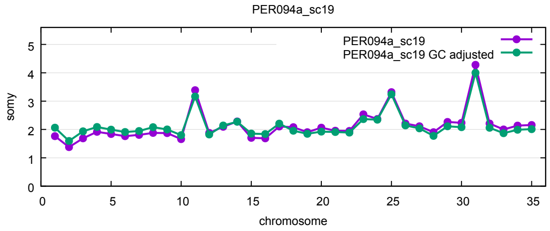

(B) PER094a_sc19

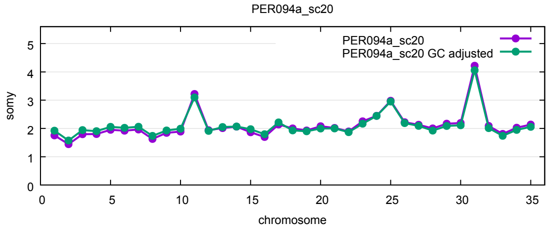

(C) PER094a_sc20

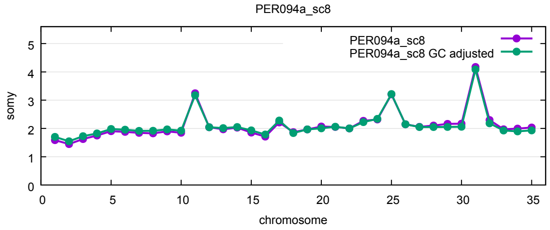

(D) PER094a_sc8

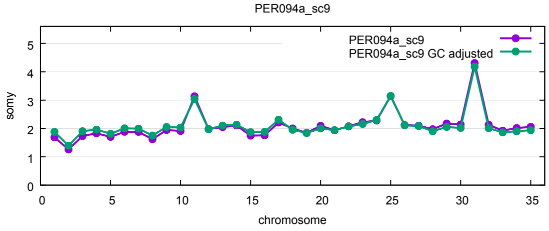

(E) PER094a_sc9

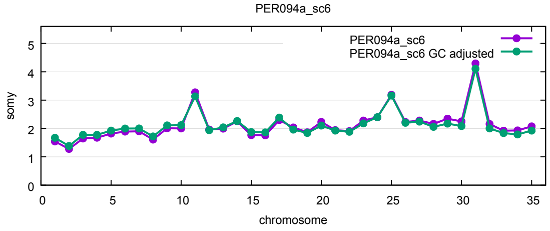

(F) PER094a_sc6

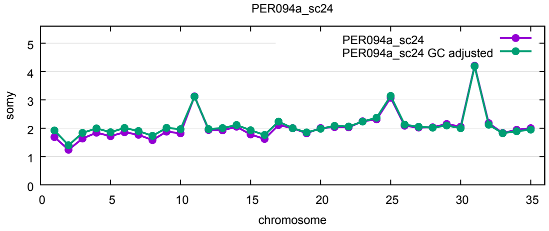

(G) PER094a_sc24

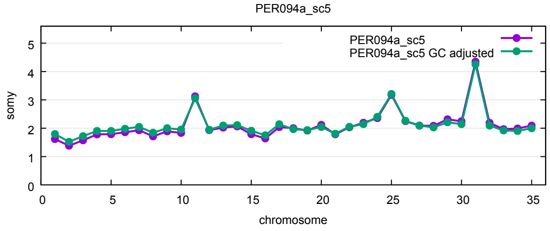

(H) PER094a_sc5

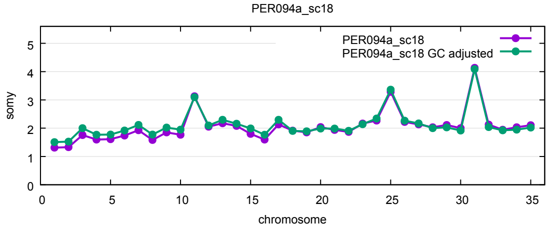

(I) PER094a_sc18

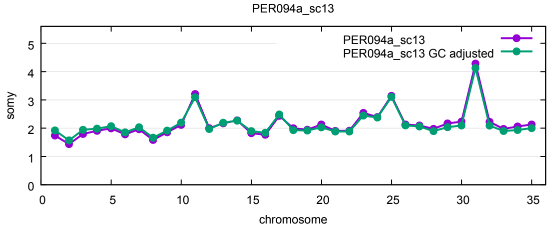

(J) PER094a_sc13

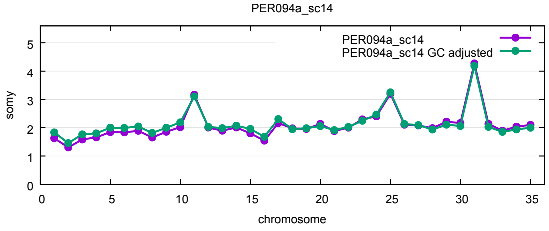

(K) PER094a_sc14

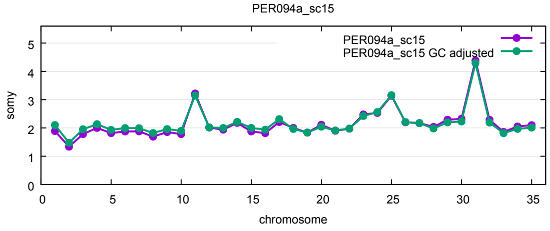

(L) PER094a_sc15

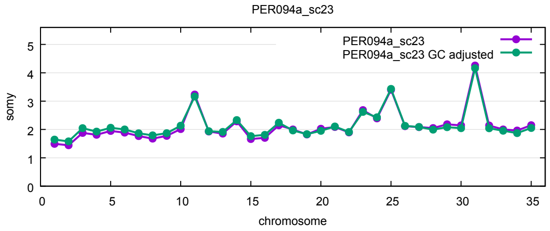

(A) PER094a_sc23

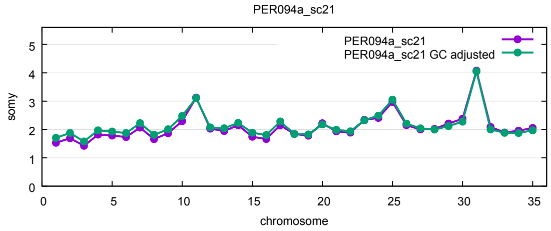

(B) PER094a_sc21

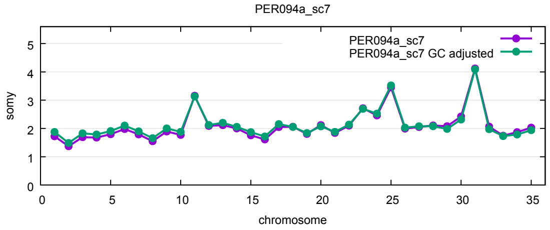

(C) PER094a_sc7

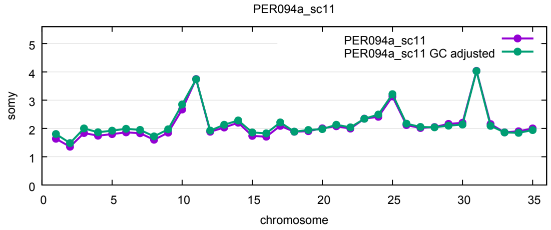

(D) PER094a_sc11

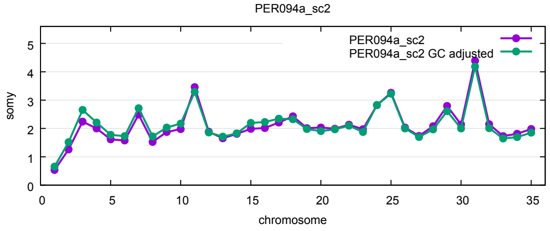

(E) PER094a_sc2

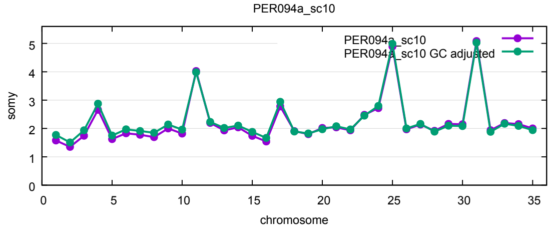

(F) PER094a_sc10

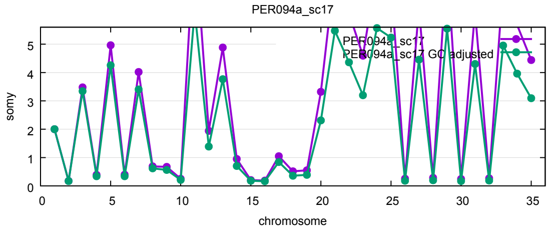

(G) PER094a_sc17

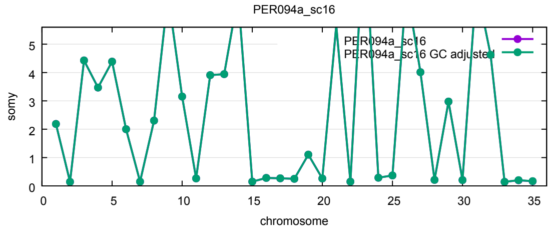

(H) PER094a_sc16

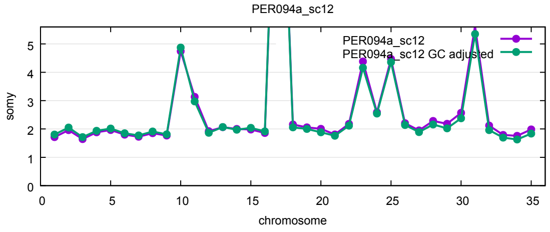

(I) PER094a_sc12

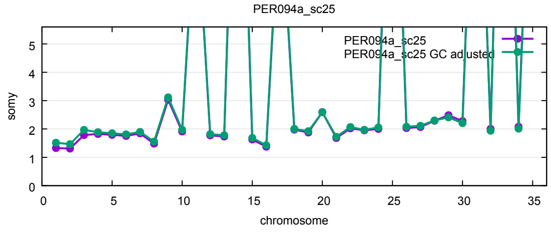

(J) PER094a_sc25

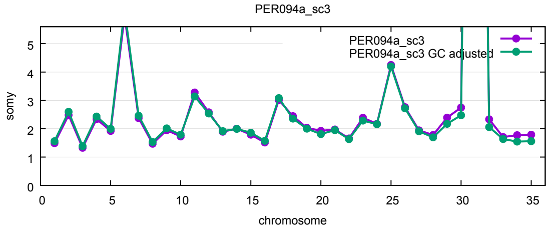

(K) PER094a_sc3

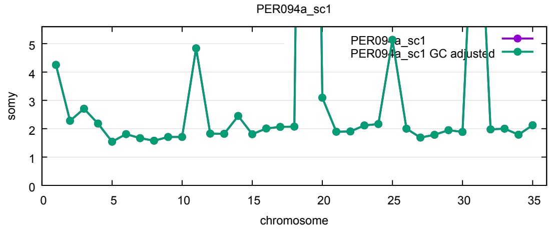

(L) PER094a_sc1

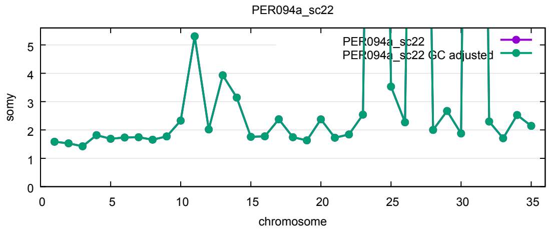

(A) PER094a_sc22

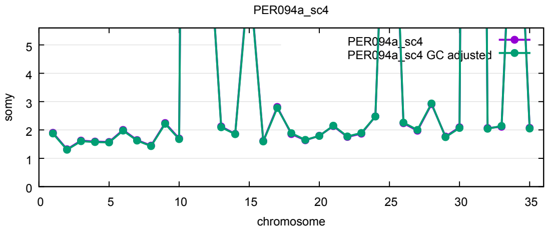

(B) PER094a_sc4
